# A novel technique to prepare a single cell suspension of isolated quiescent human hepatic stellate cells

**DOI:** 10.1038/s41598-019-49287-7

**Published:** 2019-09-04

**Authors:** Xiangyu Zhai, Wei Wang, Dandan Dou, Yunlong Ma, Du Gang, Zhengchen Jiang, Binyao Shi, Bin Jin

**Affiliations:** 1 Department of general surgery, Qilu hospital of Shandong University, Jinan, China; 2 School of medicine, Shandong University, Jinan, China; 3 School of basic medical sciences, Shandong University, Jinan, China

**Keywords:** Hepatic stellate cells, Liver diseases, Liver diseases, Hepatic stellate cells, Hepatic stellate cells

## Abstract

To explore a simple and easy-to-learn procedure for the isolation of human quiescent hepatic stellate cells (HSCs) that requires no advanced training. Thus reducing costs and increasing efficiency. This protocol will provide sufficient primary cells with minimal contaminants for future basic research on diseases associated with human HSCs. Normal liver tissues were isolated from patients undergoing hepatic hemangioma resection, and a single cell suspension of these tissues was prepared using the Gentle MACS tissue processor. By using this method, the difficulty of the procedure was reduced, fewer cells were lost during the preparation treatments, and the maximal activity of single cells was maintained. Following preparation of the cell suspension, the HSCs were further isolated using a Nycodenz density gradient. Cell viability was examined by trypan blue staining, and the purity of the quiescent human HSCs was determined by autofluorescence and oil red O staining. Activated and quiescent human HSCs were identified using immunofluorescence and Western blotting. The cell cycle distribution in activated and quiescent human HSCs was analyzed by flow cytometry.The recovery rate of the HSCs was approximately (2.1 ± 0.23) × 10^6^ of tissue, with 94.43 ± 1.89% cell viability and 93.8 ± 1.52% purity. The technique used in this study is a simple, high-yield, and repeatable method for HSC isolation that is worthy of recommendation.

## Introduction

Hepatic stellate cells (HSCs) play critical roles in the development and progression of hepatic lesions. The majority of hyperplasia of fibrous connective tissues localized in the liver interstitium is attributed to the large amount of extracellular matrix secreted by HSCs^1^. Continued improvement of the primary culture technique developed by Knook *et al*.^2^ in the 1980s has significantly increased the efficiency of *in vitro* primary culture of rat HSCs and has accelerated the discovery of the roles of HSCs in the development of benign and malignant liver diseases. Primary cell culture technology is a key component of studies of HSCs. However, many current studies are limited to quiescent HSCs of animal origin. Researchers have faced many technical difficulties in obtaining quiescent human HSCs, with the major issues including poor activity, poor yield and poor purity. Therefore, obtaining human HSCs with high purity and better efficiency using a simpler and faster procedure is an essential prerequisite for further studies on human HSCs.

Traditional cell culture methods for HSCs can be divided into two types: explant culture of tissue blocks and enzyme perfusion combined with density gradient centrifugation. Explant tissue culture is simple to perform, but the transformation from quiescent HSCs into activated HSCs cannot be efficiently controlled during culture, and limitations in the purity of the final quiescent HSCs have been difficult to overcome. Since its initial report in the 1980s, the enzyme perfusion-density gradient centrifugation method has been continuously improved and optimized. However, the enzyme perfusion process is still quite complicated. The time required for perfusion is difficult to master, and the equipment is expensive. Moreover, the method cannot be applied for smaller pieces of human hepatic tissues that lack a vascular system. In this study, we improved the current techniques commonly used to isolate HSCs and created an innovative method for the isolation and culture of quiescent human HSCs. Using the Gentle MACS tissue processor to mechanically disrupt the tissue in a gentle and stable manner is a critical step in this procedure. This procedure is a key technological improvement that optimizes the single cell suspension process. Using this procedure, the yield was greatly improved, and the cells were more active, proving that the method was more stable and efficient than traditional methods. Application of this modified method has significantly improved the isolation rate of human HSCs and can provide a reliable technical foundation for primary culture of quiescent human HSCs.

## Material

### Liver tissues

The human liver tissues used in this study were obtained from patients with liver diseases who underwent treatment in the Department of Hepatobiliary Surgery, Qilu Hospital of Shandong University. The patients selected for the study gave informed consent, and the study was approved by the ethics committee of Qilu Hospital of Shandong University. All experiments were performed in accordance with the relevant guidelines and regulations. The normal liver tissues used in our study were obtained from surgical patients with noncancerous hepatic lesions (e.g., hepatic hemangioma, etc.).

### Instruments

1. Standard laboratory equipment.
2. Precooled centrifuges (5810 R, Eppendorf, Germany)
3. Sterile surgical instruments and preparation tools
4. Sterile 15-ml plastic tubes (Biologix, 10–9501, China)
5. Sterile 50-ml plastic tubes (Biologix, 10–0501, China)
6. Pasteur pipettes (Biologix, 30–0135, China)
7. Standard cell culture room equipped with a sterile hood and a humidified incubator with 5% CO_2_ fumigation
8. Cell culture plates (Petri dishes and 6-well plates) (Biologix, 07–6006, China)
9. 70-μm cell strainer (Biologix, 15–1070, China)
10. Cell counting chambers (Biologix, 07–2104, China)
11. Inverted fluorescence microscope (NIKON, Ti-S, Japan)
12. Gentle MACS tissue processor (Miltenyi Biotec, 130-093-235, Germany)
13. Gentle MACS C Tube (Miltenyi Biotec, 130-093-237, Germany)

### Reagents

All materials must be filtered with a 0.20-μm sterile filter.

1. Enzyme H solution: Added Three milliliters of Dulbecco’s modified Eagle’s medium (DMEM) was added to the lyophilized Enzyme H powder and mixed them evenly by shaking gently. Aliquots were cryopreserved at −20 °C. Repeated freezing and thawing were avoided. (Miltenyi Biotec, 130-093-237, Germany)
2. Enzyme R solution: DMEM (2.7 ml) was added to the lyophilized Enzyme R powder. After mixing, the solution aliquots were cryopreserved at −20 °C. Repeated freezing and thawing were avoided. (Miltenyi Biotec, 130-093-237, Germany)
3. Enzyme A solution: One milliliterl of DMEM was added to the lyophilized Enzyme a powder; vigorous shaking was avoided while mixing. The aliquots were cryopreserved at −20 °C. (Miltenyi Biotec, 130-093-237, Germany)
4. DNase I (Roche, Switzerland)
5. Nycodenz Separation Solution (density 1.131 g/ml, Beijing Solarbio Science & Technology Co., Ltd.)
6. RPMI 1640 medium (Gibco, USA)
7. DMEM/F12 medium (Gibco, USA)
8. Gey’s balanced salt solution (GBSS, Sigma, USA)
9. Phosphate-buffered saline (PBS, Gibco, USA)
10. Penicillin-streptomycin (Gibco, USA)
11. Fetal bovine serum (FBS, Gibco, USA)
12. Oil red O (Sigma, USA)
13. Trypan blue (Beyotime Biotechnology, China)
14. Primary antibodies:
15. Rabbit anti-α-smooth muscle actin (SMA) monoclonal antibody (Abcam, USA)
16. Secondary antibody: goat anti-rabbit IgG (Abcam, UK).

### Methods

**Figure 1:**
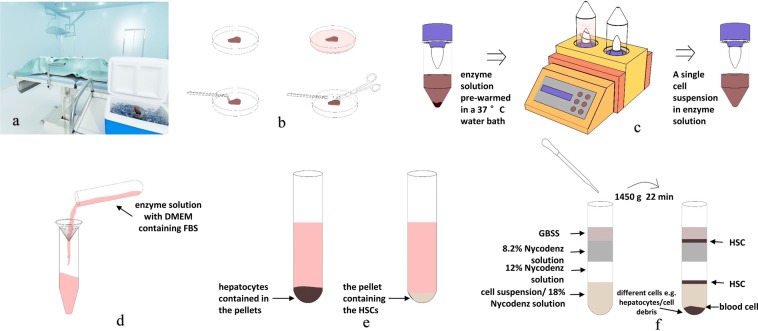
Schematic overview of the protocol for isolation of human HSC. (**a**) Surgically removed fresh normal liver tissues were processed and collected in an ice box at 4 °C under aseptic conditions. (**b**) The connective tissues, bile ducts, and blood vessels were trimmed away as much as possible using ophthalmic scissors, and the tissue were cut into small approximately 1-mm pieces. (**c**) Gentle MACSTM tissue processor disrupt the tissue in a gentle and stable setting. (**d**) Stop digestion and filtered. (**e**) Isolation human hepatic cells and human hepatic stellate cells. (**f**) Nycodenz density gradient centrifugation.

The individual steps are schematically summarized in Fig. 1

### Preparation of a single cell suspension from human hepatic tissue

**Figure 2:**
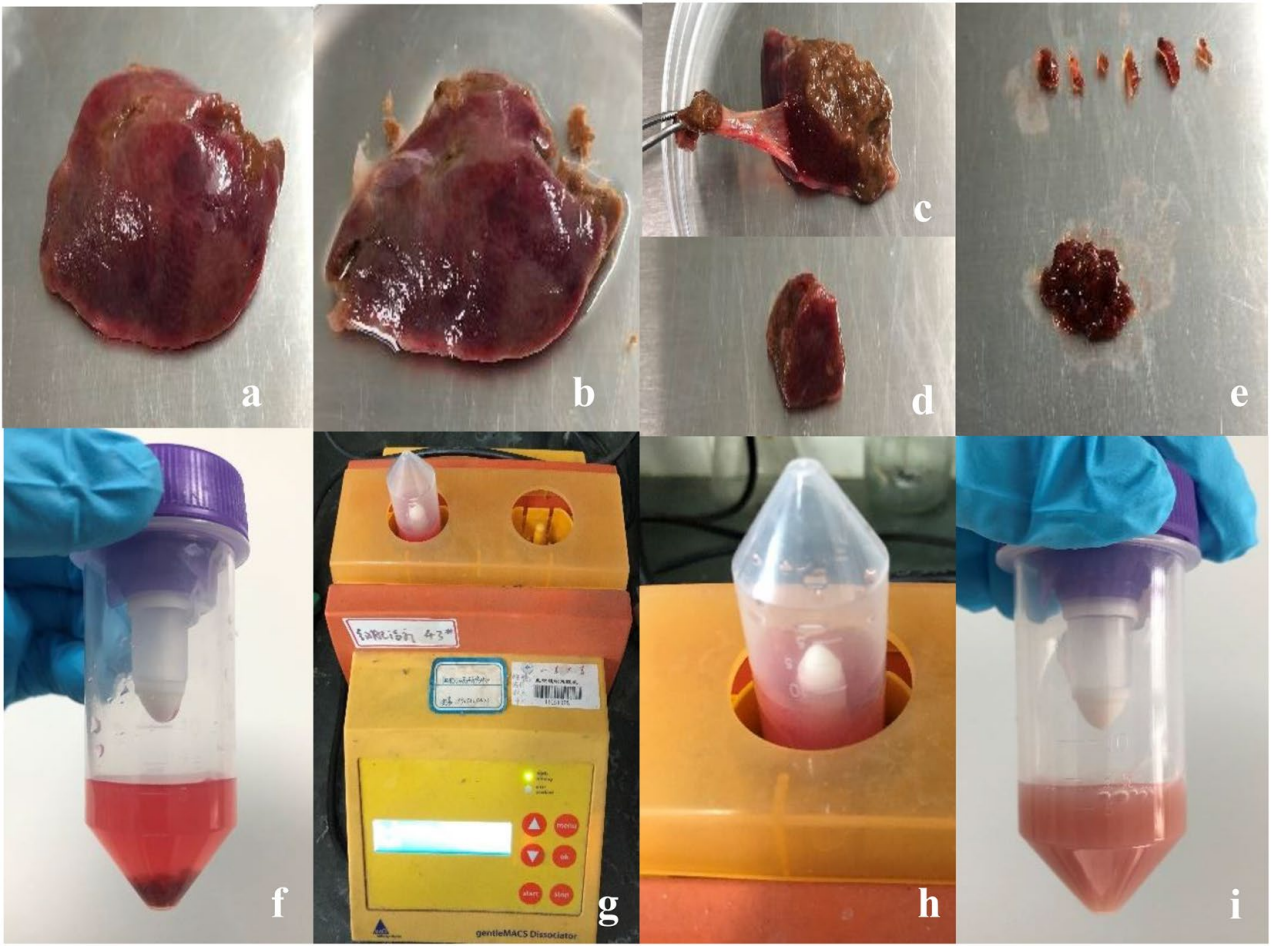
(**a**) The specimen was placed in a Petri dish. (**b**) The specimen washed with a sterile PBS solution to remove residual blood from the tissue surface. (**c**) The connective tissues were trimmed away. (**d**) The tissues were cut into blocks approximately 1 cm in diameter. (**e**) The connective tissues, bile ducts, and blood vessels were trimmed away as much as possible. These tissue blocks were further chopped into small approximately 1-mm pieces. (**f**) The mixed-enzyme solution and the liver tissue. (**g**) The Gentle MACSTM tissue processor is running the h_tumor_01.01 program. (**h**) Tissue gently digested in the C tube. (**i**) Single cell suspension of liver tissue.

Surgically removed fresh normal liver tissues were processed and collected in an ice box at 4 °C under aseptic conditions. The tissues were quickly transported to the cell culture chamber. The specimen was placed in a Petri dish and washed 5 times with a sterile PBS solution to remove residual blood from the tissue surface (Fig. 2a,b). The connective tissues were trimmed away as much as possible, and the tissues were cut into blocks approximately 1 cm in diameter (Fig. 2c,d). The bile ducts, and blood vessels were trimmed away as much as possible using ophthalmic scissors, and the tissue were blocks were further chopped into small approximately 1-mm pieces and placed into a glass Petri dish (Fig. 2e). The premixed working solution (100 μl of penicillin-streptomycin, 250 μl of Enzyme H, 150 μl of Enzyme R, and 20 μl of Enzyme A) was added to 5 ml of DMEM and mixed well to make the mixed-enzyme solution (required for 2–4 g of tissues) (Fig. 2f). To prepare the single cell suspension of human liver tissue, the mixed-enzyme solution was prewarmed in a 37 °C water bath for 10 min before adding it to the tissues. The enzymatic treatment was carried out in the GentleMACS^TM^ tissue processor at 37 °C for 15 minutes with constant shaking using the h_tumor_01.01 program (Fig. 2g,h).

### Isolation of human hepatic cells

**Figure 3:**
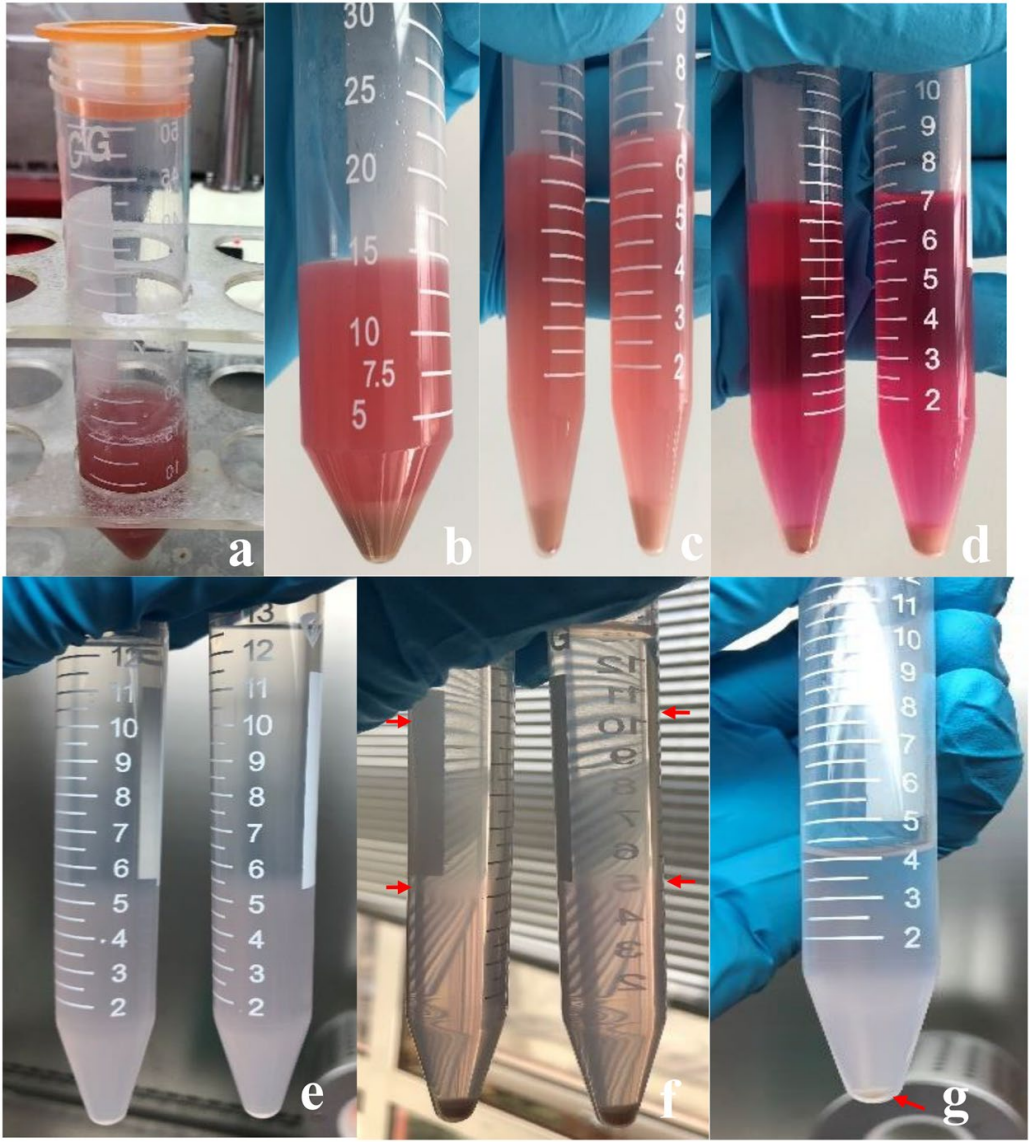
(**a**) The solution was filtered through a SmartStrainer filter. (**b**) Isolate the hepatocytes. (**c**) The pellet containing the HSCs. (**d**) DMEM washing pellet. (**e**) Preparation of 15 ml tubes for Nycodenz density centrifugation. (**f**) After density centrifugation, HSCs can be identified as a white cell layer that is floating at the surface of the gradient. (**g**) The HSC-containing layers are collected and combined, filled with GASS, and centrifuged again. After centrifugation, a white pellet is visible.

The enzymatic digestion (Fig. 2i) was stopped by diluting the enzyme solution with 10 ml of DMEM containing penicillin-streptomycin and FBS. The solution was filtered through a SmartStrainer filter (70 μm), and the filter was rinsed with DMEM containing penicillin-streptomycin and FBS after cell filtration (Fig. 3a). The filtered cell suspension was centrifuged at 50 g for 7 minutes (4 °C) to isolate the hepatocytes, which were contained in the pellets (Fig. 3b). The supernatant was centrifuged again at 450 g for 8 minutes (4 °C); then, the supernatant was discarded, and the pellet containing the HSCs was collected (Fig. 3c). The pellet was suspended in 7 ml of DMEM and centrifuged at 450 g for 7 minutes (4 °C) (Fig. 3d). The supernatant was removed as much as possible. One hundred fifty microliters of DNase solution and 10 ml of 18% Nycodenz were added sequentially to resuspend the pellet.

### Nycodenz density gradient centrifugation

Five milliliters of the cell suspension in the 18% Nycodenz solution was added to each of two 15-ml centrifuge tubes. Then, 3 ml of 12% Nycodenz and 3 ml of 8.2% Nycodenz were added gently. After further mixing with 2 ml of GBSS (Fig. 3e), the solutions were subjected to horizontal centrifugation at 1450 g for 22 min (4 °C) without braking. After the centrifugation was completed, the HSCs were aggregated at the interface of the 8.2% Nycodenz solution and the GBSS solution and the 18% and 12% Nycodenz solutions (Fig. 3f). The cells were carefully collected into another centrifuge tube and then evenly mixed in 4 ml of 1× PBS. The solution was centrifuged at 150 g for 8 min at 4 °C (Fig. 3g). The resulting supernatant was discarded, and the cell pellet was resuspended in RPMI 1640 medium containing FBS, followed by trypan blue staining and cell counting. The cell suspension was transferred to a carbon dioxide cell incubator (37 °C and 5% CO_2_) and allowed to stand still for at least 24 hours. The medium was changed every 36 hours.

### Oil red O staining

The HSC primary culture was collected. After removing the medium, the cells were washed 3 times with 1× PBS for 30 s before fixation in 1 ml of 10% paraformaldehyde solution for 15 min at room temperature. After fixation, the cells were washed with deionized water three times for 30 s per wash. Then, 3 ml of oil red O working solution was added along the wall of the well. The cells were incubated with oil red O for 30 min and then rinsed in deionized water 3 times for 30 s per wash. Mayer’s hematoxylin solution was used to stain the nuclei for 1 min, and the cells were rinsed again with deionized water for 30 s, followed by microscopic imaging.

### Immunofluorescence

Activated HSCs were seeded into 6-well plates with chambered slides and incubated at 37 °C for 48 h under 5% CO_2_. The slides were removed and washed twice with PBS before fixation in 2% paraformaldehyde-containing PBS solution for 10 min. The fixative solution was washed away with PBS 3 times for 5 min per wash. Then, the cells were treated with 0.5% Triton X-100 (in PBS solution) for 10 min to permeabilize the membrane before washing with 1× PBS 3 times for 5 min per wash. The slides were transferred into endogenous antigen blocking solution (0.75% H_2_O_2_) and blocked for 30 min in the dark. The blocking solution was replaced, and the cells were washed with PBS solution 3 times for 5 min. Primary antibodies rabbit anti-α-SMA antibody (1:100) was added, and the slides were incubated at 4 °C overnight. After 3 5-min washes with PBS, the secondary antibody (secondary antibody:PBS = 1:500) coupled with a primary anti-fluorescent dye was added, followed by 2 hours of incubation in the dark at room temperature. The slides were washed in 4, 6-diamidino-2-phenylindole (DAPI:PBS = 1:1,000) to stain the nuclei and were then sealed. The cells were observed under a fluorescence microscope, and the HSC purity was analyzed. Cell purity (%) = number of positive cells with fluorescent signals / total number of DAPI nuclear stained cells × 100%.

### Western blot analysis

For Western blot analysis, the protein was collected from freshly human HSCs and cultured for 3 days, 7 days and 10 days. The primary antibodies used was specific for α-SMA (Proteintech, US; 1:1000).

### Cell cycle

Cell cycle analysis kits were purchased from BD Pharmingen (San Diego, CA, USA). For cell cycle analysis, the cells were harvested after treatment, fixed with ice-cold 70% ethanol solution, hydrolyzed with RNase A, and stained with propidium iodide (PI) for 20 min. Cell cycle analysis was performed by a FACS Calibur flow cytometer (Becton Dickinson, San Diego, CA).

## Results

### The cell yield and viability

**Figure 4:**
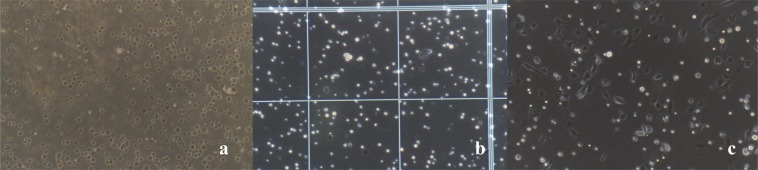
(**a**) Freshly isolated HSCs. (**b**) Trypan blue staining and cell counting. (**c**) HSCs adhere to the wall after 24 h. All images were taken at 200× magnification.

We were able to obtain approximately (2.1 ± 0.23) × 10^6^ quiescent HSCs/g of normal liver tissue. The cell viability was approximately 94.43 ± 1.89%, as determined by trypan blue staining (Fig. 4b). The cell purity was approximately 93.8 ± 1.52% as determined by oil red O staining.

### Cell identification

1. Quiescent HSCs: Freshly isolated HSCs tend to adhere to the wall after approximately 24 h (Fig. 4a,c). After adherence, the isolated cells showed typical characteristics of quiescent HSCs, including a small volume, oblate shape, and high transparency with many cytoplasmic lipid droplets that were highly refractive and gathered around the nuclei. The fat droplets in the quiescent HSCs appeared red after oil red O staining and formed strings of “oil beads” of different sizes; they wrapped around the nuclei like a flower wreath and were also dispersed in the cytoplasm (Fig. 5a,b). Quiescent HSCs also spontaneously exhibited transient blue-green fluorescence at the excitation wavelength of 328 nm under the fluorescence microscope (Fig. 5c,d). The isolated HSCs became activated after 36 hours, and the cells began to expand. Some cells displayed multi-angled pseudopods that extended out from the cell bodies. The lipid droplets in the cytoplasm disappeared gradually, and the cells began to increase in size, showing phenotypic characteristics of the activated state.

**Figure 5:**
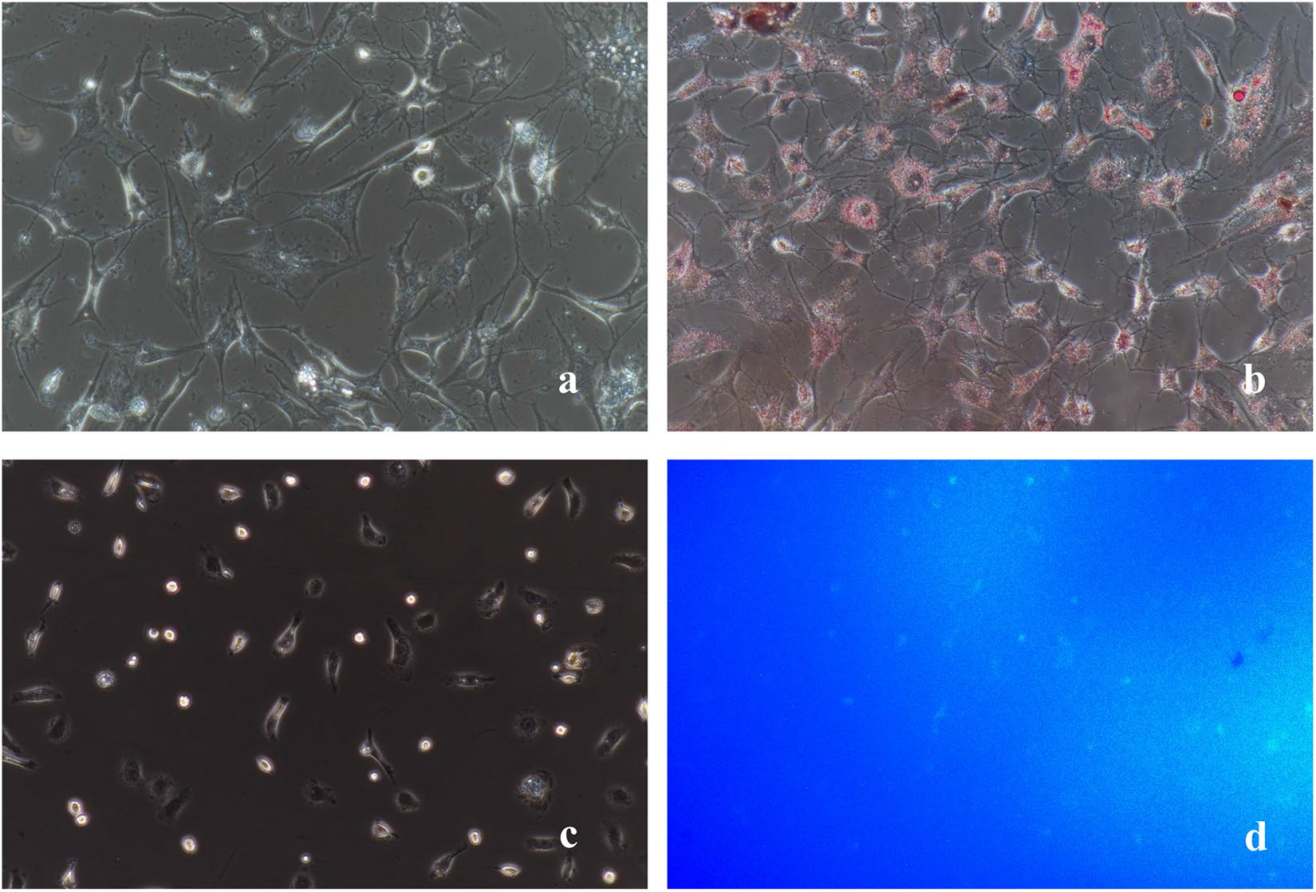
(**a**) Phase difference image. (**b**) Oil Red O. (**c**) Phase difference image. (**d**) Spontaneous blue-green fluorescence excited by ultraviolet light at 328 nm wavelength. All images were taken at 200× magnification.
2. Activated HSCs: The quiescent HSCs began to activate after 3 days in culture. Complete activation was characterized by immunofluorescence staining of specific activation marker, α-SMA (Fig. 6). Activated HSCs marker α-SMA was differentially expressed in Western blotting at different time points (0 day, 3 day, 7 day, 10 day) (Fig. 7). We evaluated the cell cycle distribution of freshly isolated HSCs and activated HSCs by flow cytometry. Compared with the activated HSCs, freshly isolated HSCs showed a significant decrease in the percentage of cells in the S phase, and the cell cycle was significantly blocked at the G1 checkpoint (Fig. 8).

**Figure 6:**
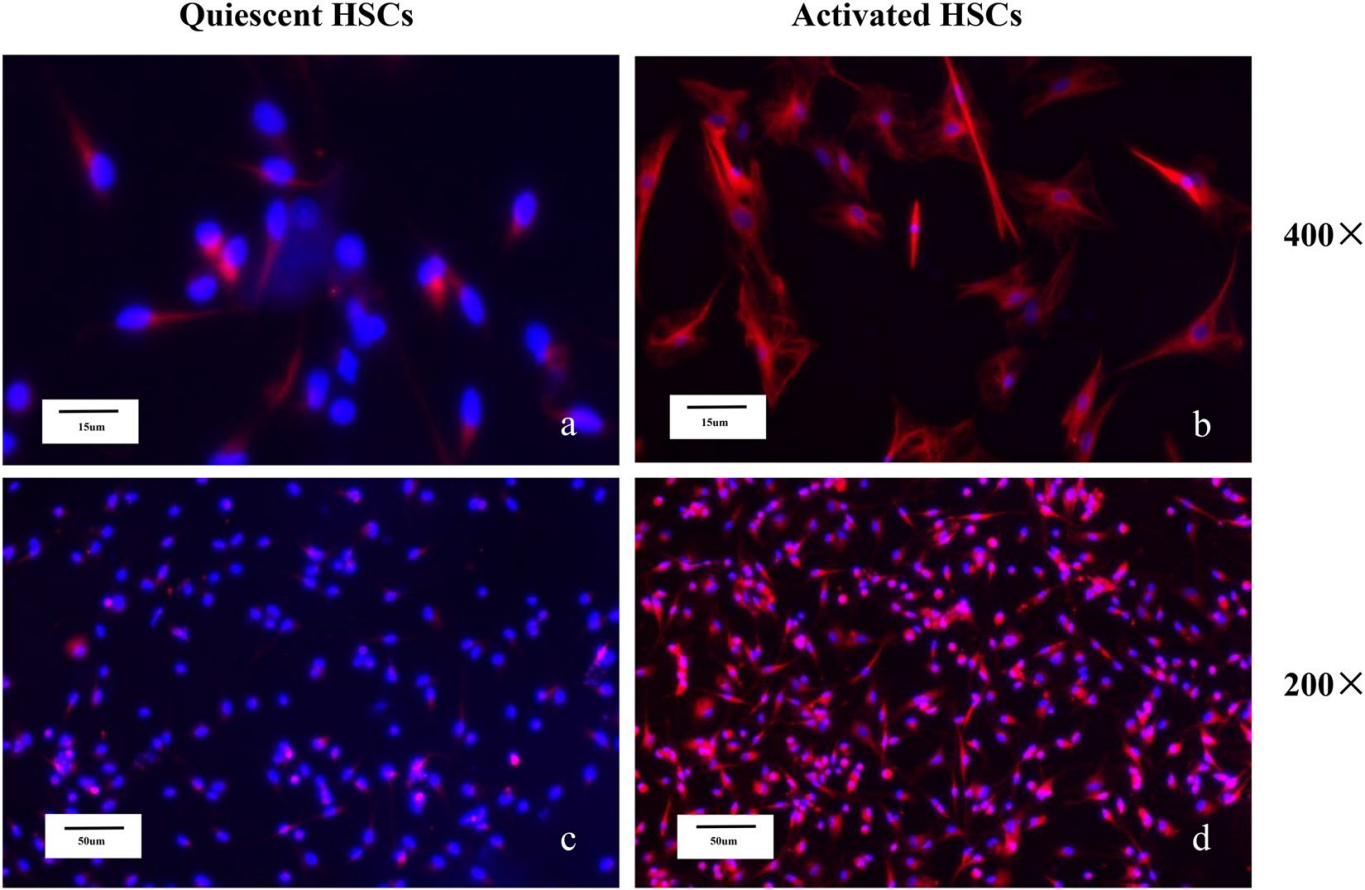
Quiescent HSCs began to activate: (**a**,**c**) α-SMA immunocytochemistry (red) of HSCs. Activated HSCs: (**b**,**d**) α-SMA immunocytochemistry (red) of HSCs. Images were taken at 200× , 400× magnification.

**Figure 7:**
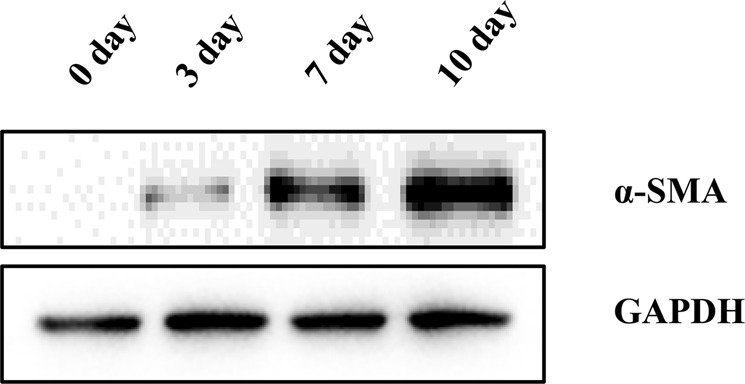
The α-SMA was differentially expressed in Western blotting at different time points (0 day, 3 day, 7 day, 10 day).

**Figure 8:**
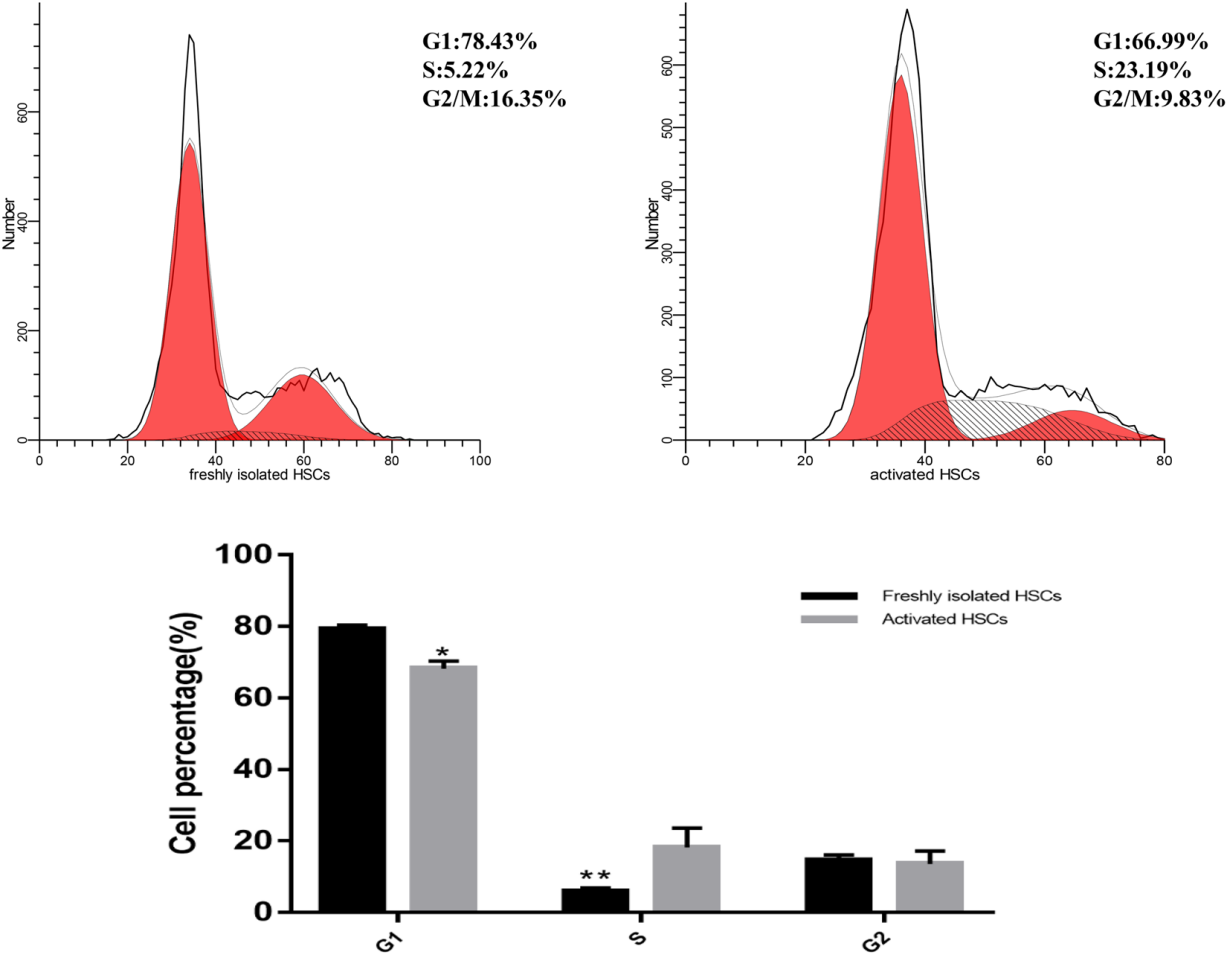
The cell cycle distribution in freshly isolated HSCs and activated HSCs were analyzed by flow cytometry.

## Discussion

HSCs are non-parenchymal cells in the liver that are located in the perisinusoidal space (Disse space) and are adjacent to hepatocytes and sinusoidal endothelial cells. Between 8 and 12 µm in size, these cells possess copious pseudopods that surround the sinusoids^3^ and account for 5%~8% of total liver cells^4^. HSCs have two phenotypes: quiescent and activated. Quiescent HSCs are the major type of cells for vitamin A storage^5^ and synthesize extracellular matrix proteins to maintain homeostasis of the sinusoidal microenvironment^6^. Other physiological functions of HSCs include supporting sinusoidal endothelial cells and regulating sinusoidal blood flow^7^. When the liver is damaged by various hepatotoxic factors, cytokines and inflammatory mediators are produced by activated immune cells and Kupffer cells, which further transform the HSCs from the quiescent to the activated state. The morphology of HSCs changes drastically after activation; the lipid droplets in the cytoplasm disappear, the characteristic protein α-SMA is expressed, and microfilaments and microtubule structures start to appear in the cytoplasm with markedly increased Golgi and rough endoplasmic reticulum development in the cells^8,9^. Accompanying these events, changes in the biological behavior of the cells also occur, such as altered cell proliferation, mobility, and contractility^9,10^. The HSCs begin to secrete various growth factors, inflammatory factors, and extracellular matrix materials^11^ as part of the repair process for liver damage^12^. More in-depth studies are being carried out on the role of HSCs in the formation and development of liver fibrosis^13,14^. Studies on the function of HSCs in liver development, regeneration, injury, and neoplastic diseases have also received increasing attention in recent years^15^. Cell isolation and cell culture techniques are important tools for accurate examinations of the function of a single cell type, and therefore, the ability to isolate and cultivate stable HSCs from the liver is essential for studies of their structure and function at the cellular level.

Knook *et al*.^2^ was the first study to successfully isolate rat HSCs by chain protease/collagenase perfusion combined with density gradient centrifugation. After nearly 30 years of application and improvement, this method is still commonly used to isolate HSCs from rats. However, in the case of human HSCs, normal hepatic tissue is often only available in very small quantities due to the small surgical margin, and thus, finding large blood vessels to perform chain protease/collagenase perfusion is difficult. Therefore, explant cultures of liver tissue blocks combined with autofluorescence sorting or flow cytometry and immunomagnetic bead sorting are more frequently used methods for the isolation of primary human HSCs.

Autofluorescence sorting is based on the presence of lipid droplets in quiescent HSCs, which spontaneously exhibit blue-green fluorescence at specific excitation wavelengths. These cells are isolated from the other liver cells by their fluorescence signals. In 1989, Matsuura *et al*.^16^ successfully isolated rat HSCs using this method. The advantage of autofluorescence sorting is that it can obtain HSCs with ultra-high purity. However, this method is limited by its high cost, complicated operational process, and significant loss of cells. Flow cytometry and immunomagnetic bead sorting are methods based on the specific surface antigens of HSCs, and the cells are screened by immunological binding. The HSCs sorted by this method show high purity, but the process is complicated and expensive. Cell viability is also greatly affected after sorting, and there is a significant loss in cell numbers. Furthermore, this method can only isolate activated HSCs. Therefore, this method has not been widely adopted.

Based on these previous approaches, our study has made improvements and developed an innovative method. We used a tumor dissociation kit to digest normal liver tissue with appropriate enzyme solutions at the optimal concentrations. The tissues were subsequently processed gently by standardized mechanical disruption using the GentleMACS^TM^ tissue processor. This step largely avoided the extensive cell disruptions caused by excessive manual mechanical disruption and allowed us to obtain a highly active single cell suspension from the hepatic tissue using a fast, stable, efficient, and standardized approach. Density differentiation solutions (such as Nycodenz, Metrizamide, Stractan, Percoll, and Optiprep) have been used to isolate HSCs from liver tissue based on different cell densities. Quiescent HSCs contain a large amount of lipid droplets, and thus, they have the lowest density among all hepatic cells (approximately 1.053 ± 0.007) and can be easily purified and isolated from other cells in the liver through centrifugation. This method is cost-effective, practical, reliable, and highly repeatable.

Density gradient centrifugation is an important step in the purification of HSCs. The solution selected for density gradient centrifugation directly affects the purity of the isolated HSCs. Commonly used solutions for density gradient centrifugation are Nycodenz^17^, Metrizamide^2^, Stractan^18^, and Percoll^19^. Nycodenz was used in this study because of its simple configuration, low toxicity, stability, and ability to easily wash off the cells. In addition, the low cost and convenience of freshly made preparations are great advantages of this solution. Sedimentation and floatation are two widely used gradient density centrifugation methods, and both can be used to isolate and purify cells. During the sedimentation process, HSCs tend to adhere to other cells when they pass through the density barrier, resulting in a decrease in yield^20^. In our study, we found that in addition to cell loss, HSCs obtained by the sedimentation method tended to be more contaminated with cellular debris, which decreased the cell purity. This problem may be associated with the super-low density of the fragmented cell debris, which causes the cells to be unable to precipitate. Therefore, in this study, we used the cell flotation method to further improve the purity of the cells and ensure a good yield.

One study has shown that HSCs at the initiation stage of activation (initiation) differ from HSCs at the stage of complete activation (perpetuation) in terms of both cell structure and secretory function^15^. Compared with freshly isolated HSCs, activated HSCs have a strong proliferation ability. The α-SMA is expressed negatively in rat quiescent cells^21^; However, it expressed positively in the activated stellate cells^22,23^. In normal adult human liver, stellate cells are α-SMA negative^24^; stellate cells in normal embryonic and infant liver, and activated stellate cells in pathological livers or in culture are clearly α-SMA positive^25–27^. These findings suggest that HSCs at the two different activation stages undergo different protein synthesis and secretion processes, which means that HSCs at different activation phases require different identification methods. Freshly isolated quiescent HSCs can be primarily identified by cell autofluorescence after excitation with 328 nm UV light^9,28,29^^,^ or through specific oil red O labeling^30^ of triglyceride droplets in these cells, which makes HSCs easily distinguishable from other cells under a light microscope^30^. Once the HSCs reach the active phase, the cytoplasmic lipid droplets will disappear gradually, and oil red O staining is no longer suitable to identify the cells. At this stage, HSCs express specificity marker α-SMA, and thus, immunofluorescence signals and Western blotting become more reliable methods for the identification of activated HSCs.

## Conclusion

The previously available methods to isolate primary quiescent HSCs have limitations. The enzyme perfusion-Gentle MACS tissue processor-density gradient centrifugation method created in this study has great advantages in the following areas: convenient operation, strong repeatability, economic efficiency, standardized procedure and high purity of isolated cells. Thus, this technique can be widely introduced and adopted in human HSC studies and other fields.
